# Change of the vaginal microbiome with oral contraceptive therapy in women with polycystic ovary syndrome: a 6-month longitudinal cohort study

**DOI:** 10.1186/s12916-023-03196-9

**Published:** 2023-12-01

**Authors:** Xiang Hong, Pengfei Qin, Liting Gao, Lingling Huang, Yong Shi, Danhong Peng, Bei Wang

**Affiliations:** 1 Key Laboratory of Environmental Medicine and Engineering of Ministry of Education, Department of Epidemiology and Health Statistics, School of Public Health, Southeast University, Nanjing, Jiangsu China; 2 The Affiliated Obstetrics and Gynecology Hospital with Nanjing Medical University, Nanjing Women and Children’s Healthcare Hospital, Nanjing, Jiangsu China; 3 School of Medicine, Southeast University, Nanjing, Jiangsu China; 4 Department of Obstetrics and Gynecology, Zhong Da Hospital, Southeast University, Nanjing, Jiangsu China

**Keywords:** Polycystic ovary syndrome, Vaginal microbiome, Cohort study, Change trajectory

## Abstract

**Background:**

The association between the vaginal microbiome and polycystic ovary syndrome (PCOS) is reported, but the longitudinal changes in the vaginal microbiome that accompany oral contraceptive therapy have not been described.

**Methods:**

This cohort study included 50 PCOS patients who wanted to make their menstrual periods more regular and accepted only oral contraceptive therapy and lifestyle coaching, then they were successfully followed up for 6 months. Venous blood was collected, and follicle-stimulating hormone (FSH), luteinizing hormone (LH), total testosterone (T), anti-Müllerian hormone (AMH), and estradiol (E2) were assayed at baseline and at months 3 and 6. Vaginal swabs were collected at baseline and at months 3 and 6. 16S rRNA genes were sequenced to identify the microbiota structure. Latent class trajectory models were used to explore the trajectory of the changes in *Lactobacillus* abundance.

**Results:**

At 3 months, all patients reported regular periods, and the improvement lasted until 6 months. The body mass index and waist-to-hip ratio decreased with treatment (*P* < 0.01), and the AMH and T levels showed downward trends. We did not find a statistically significant relationship between hormone levels at the previous time point and the vaginal microbiota at subsequent time points (*P* > 0.05). The relative abundance of *Lactobacillus* increased with treatment, and trajectory analysis revealed five classes of *Lactobacillus* changes. Class 1, stable high level, accounted for 26%; class 2, decrease followed by increase, accounted for 18%; class 3, stable low level, accounted for 10%; class 4, increase, accounted for 20%; class 5, increase followed by decrease, accounted for 26%. Logistic models showed that compared to class 1, a higher baseline T level was associated with a reduced risk of class 2 change (odds ratio (OR) = 0.03, 95% confidence interval (CI):0.01–0.52) and class 4 change (OR = 0.10, 95% CI:0.01–0.93).

**Conclusions:**

The abundance of *Lactobacilli* increased with PCOS treatment; however, the trajectory was inconsistent for each individual. Evidence of the effects of female hormone levels on the vaginal microbiome is insufficient.

**Supplementary Information:**

The online version contains supplementary material available at 10.1186/s12916-023-03196-9.

## Background

Polycystic ovary syndrome (PCOS) affects approximately 10% of reproductive-age women with many symptoms, including hyperandrogenism and oligomenorrhea, often accompanied by obesity and acne [1]. Although there is no clear cause among many potential etiologies, an association between vaginal microbiota and PCOS has been considered in recent years [2]. Unlike the intestinal microbiota, the vaginal microbiome has a simpler structure and is thought to be related to female hormone levels [3] thus, it has potential as a PCOS biomarker [4]. In general, *Lactobacillus* is considered as the dominant genus of healthy vaginal microbiome, which plays an important role in regulating the pH of the vaginal microenvironment and controlling inflammation [5]. Previous case-controlled studies have shown that *Gardnerella vaginalis*, *Prevotella*, and *Mycoplasma hominis* are more abundant in the vaginal microbiota of PCOS patients [6]. Lu et al. found that vaginal *Streptococcus* was significantly negatively correlated with follicle-stimulating hormone (FSH) [7], and our previous study also found that testosterone level was associated with the relative abundance of *Lactobacillus iners*. [8] However, current evidence is from retrospective studies, which limits the investigation of causality and is thus not enough to provide support for clinical diagnosis or treatment of PCOS patients.

The current academic consensus is that PCOS treatment should focus on the management of the condition, which means that there is no curative treatment. Lifestyle changes are universally recommended. Oral contraceptive therapy with combination birth control pills is suitable for regulating female periods, and some assisted reproductive treatments are suggested for patients who want to become pregnant [9]. Although some data have shown that the vaginal microenvironment of women was poorer than that of healthy women [4, 6], the detection of vaginitis is not routinely recommended by current guidelines. In theory, if there is a relationship between vaginal microbiota and PCOS, the vaginal microenvironment would be improved by PCOS treatment without antibiotic intervention. However, there is no evidence to support this view.

In addition, some novel vaginal microbiota interventions have provided new possibilities for the treatment of PCOS, such as vaginal microflora transplants [10] and vaginal probiotics [11]. We conducted a cohort study focusing on women with PCOS who were administered oral contraceptive therapy for six months to reveal longitudinal changes in the vaginal microbiome. We hypothesized that the change in hormone levels would affect the vaginal microbiome and push them in a better direction. This study aimed to provide more compelling evidence for the association between PCOS and the vaginal microbiome, and new perspectives on the treatment of PCOS and vaginitis.

## Methods

### Study population

At baseline, we included women who were initially diagnosed with PCOS between March 2019 and May 2020 at the reproductive clinic of the Zhongda Hospital (Nanjing, China). The PCOS diagnosis followed the current Chinese Guidelines for Diagnosis of PCOS based on the improved Rotterdam criteria [12]. All women had at least two of three symptoms: oligo- and/or anovulation (fewer than eight cycles per year or more than three months without menstruation), clinical and/or biochemical signs of hyperandrogenism (hirsutism with a modified Ferriman-Gallwey score of ≥ 5 [12] or total testosterone > 1.77 nmol/L), and polycystic ovaries (12 or more follicles in each ovary measuring 2–9 mm in diameter and/or increased ovarian volume (> 10 mL) determined by ultrasound examination). Eighty-nine patients were included, as previously described [8]. Patients with clinical symptoms of vaginitis that required treatment (*n* = 26; 29.2%) were excluded. The remaining individuals were clinically asymptomatic and none of them had gynecological washings prior to vaginal swab collection. Then another 3 patients want to prepare for pregnancy were also excluded. Thus, patients who desired regular menstrual periods and accepted only oral contraceptive therapy and lifestyle coaching (*n* = 60, 67.4%) were followed up. Eventually, only fifty women (83.3%) completed the 6-month follow-up and without gynecological intravaginal irrigation during the study period. As a preliminary observational study design, we do not know the specific time point when the vaginal microbiota begins to change after taking the drug, thus vaginal swab samples were collected at 3 and 6 months according to the clinical practice habits. Oral contraceptives are usually required to be taken continuously for 3–6 cycles [13], and the patients would be asked to return to the hospital for disease assessment and the next course of drugs after 3 cycles [14]. Ultimately, 50 patients completed 6 cycles of treatment and completed the sampling procedure. All the participants provided written informed consent. This study was approved by the Ethics Committee of Zhongda Hospital (2018ZDSYLL072-P01).

### Patient evaluation

Baseline demographic information regarding age, marital status, and education level was collected using a standard questionnaire. Height, weight, waist circumference, and hip circumference were measured, and the body mass index (BMI, kg/m^2^) and waist-to-hip ratio (WHR) were calculated by a gynecologist. Following standard Chinese guidelines [15], patients were classified as having normal BMI (< 23.9 kg/m^2^) or a BMI indicative of overweight/obesity. Two vaginal swabs were collected by a gynecologist at the time of enrollment. One swab was used to assess the Nugent score based on standard procedures and the other was transferred to a – 80 ℃ refrigerator without any buffer for storage, then they were used for DNA extraction and 16S rRNA gene sequencing uniformly. Venous blood was collected for the determination of FSH, luteinizing hormone (LH), total testosterone (T), anti-Müllerian hormone (AMH), and estradiol (E2) by chemiluminescent immunoassays (DXI800 Immune Analyzer; Beckman Coulter Inc; Pasadena, CA. USA).

PCOS treatment focuses on patient management following clinical guidelines including assisted reproductive treatment, anti-androgens, insulin-sensitizing agents, and oral contraceptive strategies [9]. Promotion of healthy lifestyle habits includes reduced-calorie diets, weight loss, and increased physical activity [16]. We focused on patients who wanted regular menstrual periods and accepted oral contraceptive therapy alone for six cycles (*n* = 60, 67.4%). According to the Chinese clinical guidelines, we chose drospirenone and ethinylestradiol tablets (Yasmin, Bayer Pharma AG) as the therapeutic agents in this group of patients. During the treatment period, the patients took one tablet (3 mg drospirenone and 0.03 mg ethinylestradiol) per day, continuously for 21 days, and then waited for menstruation. On the second day of the menstrual period, start the next round of 21 consecutive days of medication. Meanwhile, to satisfy the ethical requirements, the health education for healthy lifestyle was necessary for every PCOS patient. The patients returned for follow-up at 3 and 6 months to evaluate the clinical symptoms and menstruation. Hormone levels were reassessed in the early follicular phase. In month 3, we required that the blood samples should be collected on the second days of the menstrual period without drug intake. Only after laboratory tests are completed can the patient proceed to the next round of prescription drugs from the doctor. In month 6, the patients had finished the treatment process. So the oral contraceptive would not directly impact the hormone levels. The vaginal swabs were collected after menstruation, it is usually on the seventh day. The assays and instruments were the same at baseline and follow-up.

### DNA extraction and 16S rRNA gene sequencing

The frozen swabs were eluted with phosphate-buffered saline, and TIANamp bacterial DNA kits (Tiangen Biochemical Technology, Beijing, China) were used to extract and purify the nucleic acids. DNA was extracted, as previously described [17]. Using universal primers (338F:5′-ACTCCTACGGGAGGCAGCA-3′, 806R:5′-GGACTACHVGGGTWTCTAAT-3′), the V3–V4 region of the 16S rRNA gene was amplified by polymerase chain reaction and then sequenced on an Illumina HiSeq 2500 platform (Beijing Biomarker Technologies Co. Ltd. Beijing, China). Negative control swabs (blank) were processed alongside each DNA extraction set. No amplicons were observed following PCR and gel electrophoresis, thus these were not subsequently sequenced.

The sequencing data were processed as previously described [17]. Briefly, paired-end reads were merged, and tags of low quality, with more than six mismatches compared with the primers, average quality scores of < 20 in a 50 bp sliding window, or shorter than 350 bp, were removed using FLASH (v1.2.7) and Trimmomatic software (v0.33). Sequences were then clustered into operational taxonomic units (OTUs) at a 97% similarity level with USEARCH (version 10.0) [18]. After sequence clustering, the Silva dataset (v138.1) was used to annotate taxonomic information using QIIME software. The number of reads for each sample was normalized to that of the sample with the smallest sequence (*n* = 41,439). OTUs with < 10 copies in individual samples were excluded for the reliability and accuracy [19]. A sensitivity analysis was performed based on the amplicon sequence variants (ASVs) with the DADA2 method [20] through QIIME2 software. The raw sequencing data is stored in the NCBI platform [21]. Bioinformatics annotation was performed using the Biomarker BioCloud platform (www.biocloud.org).

### Statistical analysis

Α-diversity indices, including the Shannon and Simpson indices, were calculated using mothur software (version 1.30) based on the OTU data. A higher Shannon index was associated with a more diverse and rich vaginal microbiome [22]. The relative abundance of OTUs was calculated, then Aitchison’s log-ratio transformation [23] was used for this compositional data for further analysis [24]. β diversity between groups was based on binary Jaccard and Bray–Curtis distances, and that was reported using principal coordinate analysis (PCoA) plots. Permutational multivariate analysis of variance (PERMANOVA) was used to explore the potential associations between different hormone indicators and the vaginal microbiome, adjusting for potential confounding factors (age, marital status, education level, BMI and WHR), using the adonis2 function in R package *vegan* (v 2.6–2). Linear discriminant effect size (LEfSe) analysis was used to determine potential biomarkers (LDA > 4.0). Meanwhile, in order to control the false positive rate and solve the sparse data problem, multivariable association with linear models (MaAslin2) was also used to compare the abundances of different species among groups, with the *Maaslin2* package (version 1.8.0) [25]. The association of bacterial genera with hormonal changes was estimated using Spearman’s correlation coefficients, the obtained results were imported into Cytosape 3.9.1 software for visualization, and its built-in “Network Analyzer” tool module was used for topological network analysis.

Considering the non-independence of the test results from the three visits, we used mixed-effects models to explore the changes in continuous variables at different times. To explore the differences among individuals, we used latent class trajectory modeling (LCTM) for hormone indices and the relative abundance of *Lactobacillus*. The *lcmm* package (version 2.0.0) in R was used followed by the standard framework. Briefly, the chosen number of classes was based on the lowest Bayesian information criterion [26]. Multinominal logistic regression was used to estimate the odds ratios (ORs) and their 95% confidence intervals (CIs) for different classes after adjusting the potential confounding factors, including age, marital status, education level, BMI, and WHR. All analyses were performed with R software (version 4.1.3). Two-sided *P* values of < 0.05 were deemed to be statistically significant. In order to control the false discovery rate for multiple hypothesis testing, the Benjamini–Hochberg method was used to reduce the statistical type I error.

## Results

### Baseline characteristics of participants

Of the 60 participants who met the inclusion criteria, 50 completed three follow-up visits and their vaginal swabs were collected. At baseline, the average age was 26.0 years (SD = 4.51), ranging from 20 to 38 years old. 56% were married, 64% had an educational level of graduate or above, 38% were overweight or obese, and the average WHR was 0.82. Details are presented in Table 1.

**Table 1:** Participant baseline characteristics

	*n*	%
Age, mean (SD), year	26.0 (4.51)	
Marital status		
Married	28	56.0
Unmarried	22	44.0
Educational level		
Graduate and above	32	64.0
High school diploma or below	18	36.0
BMI, mean (SD), kg/m^2^	22.8 (4.01)	
Normal	31	62.0
Overweight/obesity	19	38.0
WHR, mean (SD)	0.82 (0.04)	

### Clinical treatment effectiveness and longitudinal changes of hormone levels

**Fig. 1:**
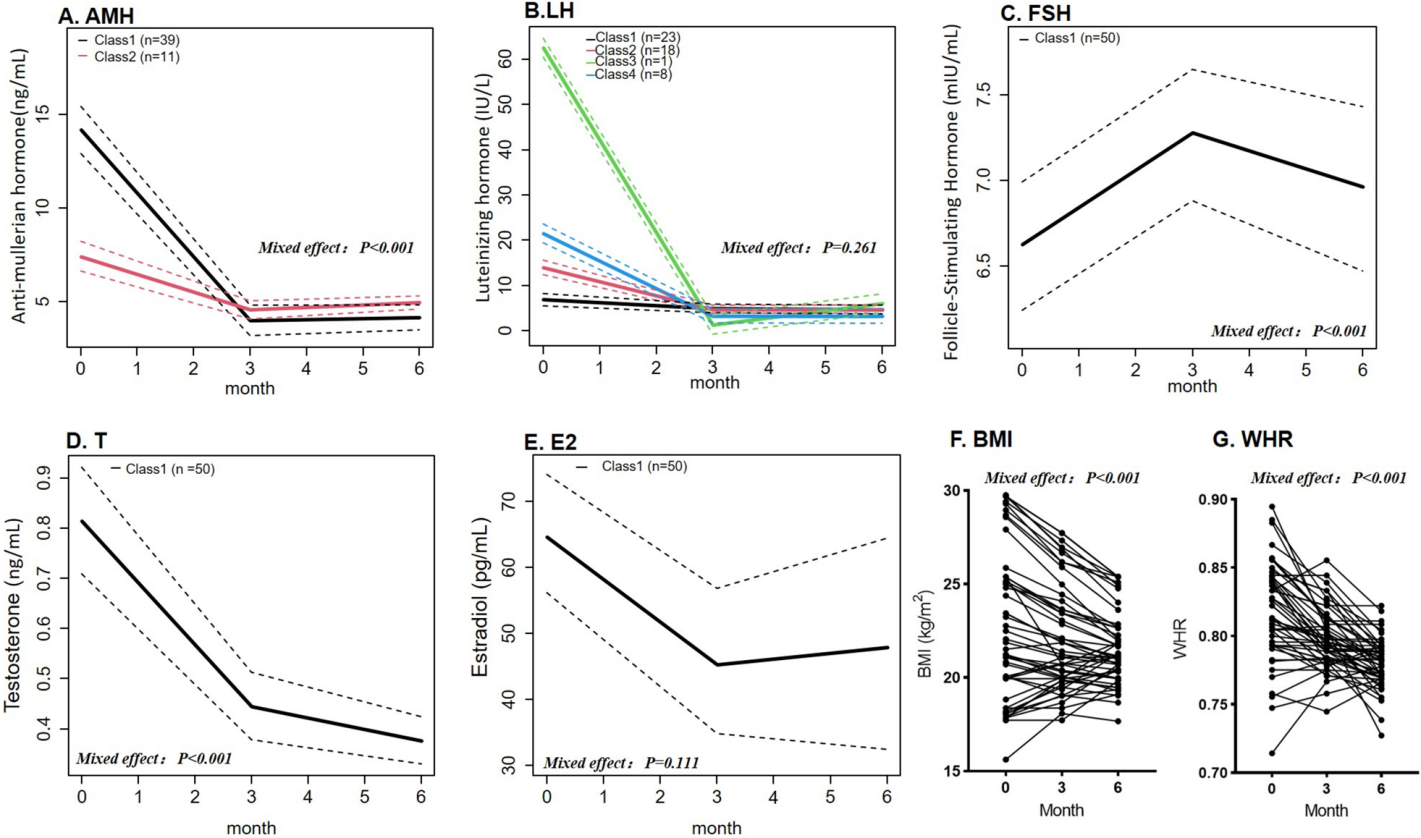
Longitudinal changes of hormone levels and anthropometry. **A**–**E** Changes in anti-Müllerian hormone (AMH), luteinizing hormone (LH), follicle-stimulating hormone (FSH), total testosterone (T), and estradiol (E2) concentration. Latent class trajectory models were used to explore differences in their trajectories Dotted lines show confidence intervals. **F** Body mass index (BMI). **G** Waist-to-hip ratio (WHR). All *P*-values are from mixed effect models

At 3 months, all patients reported regular periods, and the improvement lasted until 6 months. The changes in hormone levels were significant (Additional file 1: Table S1). Changes in AMH levels (Fig. 1A) during the 6-month follow-up were significant (*P* < 0.001). In the first 3 months, the level declined rapidly from a median of 8.41 ng/mL to 4.44 ng/mL. The LCTM analysis revealed that the changes were clustered into two classes. Most patients (*n* = 39, 78%) were classified as class 1 with a higher baseline AMH level. The overall decline in LH levels (Fig. 1B) was relatively small, and the mixed-effect test was not significant (*P* = 0.261). In the LCTM analysis, four latent classes were dependent on the baseline LH level. There was little difference between the second and third visits. The FSH level increased slightly but not much (mixed-effect *P* < 0.001, median levels were 6.65 vs. 7.39 vs. 7.32 mIU/mL, Fig. 1C). T declined with the treatment (mixed-effect *P* < 0.001, median values 0.74 vs. 0.45 vs. 0.35 ng/mL, Fig. 1D). The decrease in E2 levels was not significant (mixed-effect *P* = 0.111, Fig. 1E). All patients had similar patterns of change in FSH, T, and E2 levels because the LCTM analysis found only one class. However, the trajectory graphs (Fig. 1C–E) show that the CIs of the levels of the three indices were wide.

Most patients showed a healthy weight management trend. As shown in Fig. 1F, most women with high baseline BMIs lost weight, and their BMI declined. A few women with lower baseline BMIs showed an upward trend in body weight. The overall change was statistically significant (*P* < 0.001). Figure 1G shows that the change in WHR followed a downward trend (*P* < 0.001).

### Longitudinal changes in the vaginal microbiome

**Fig. 2:**
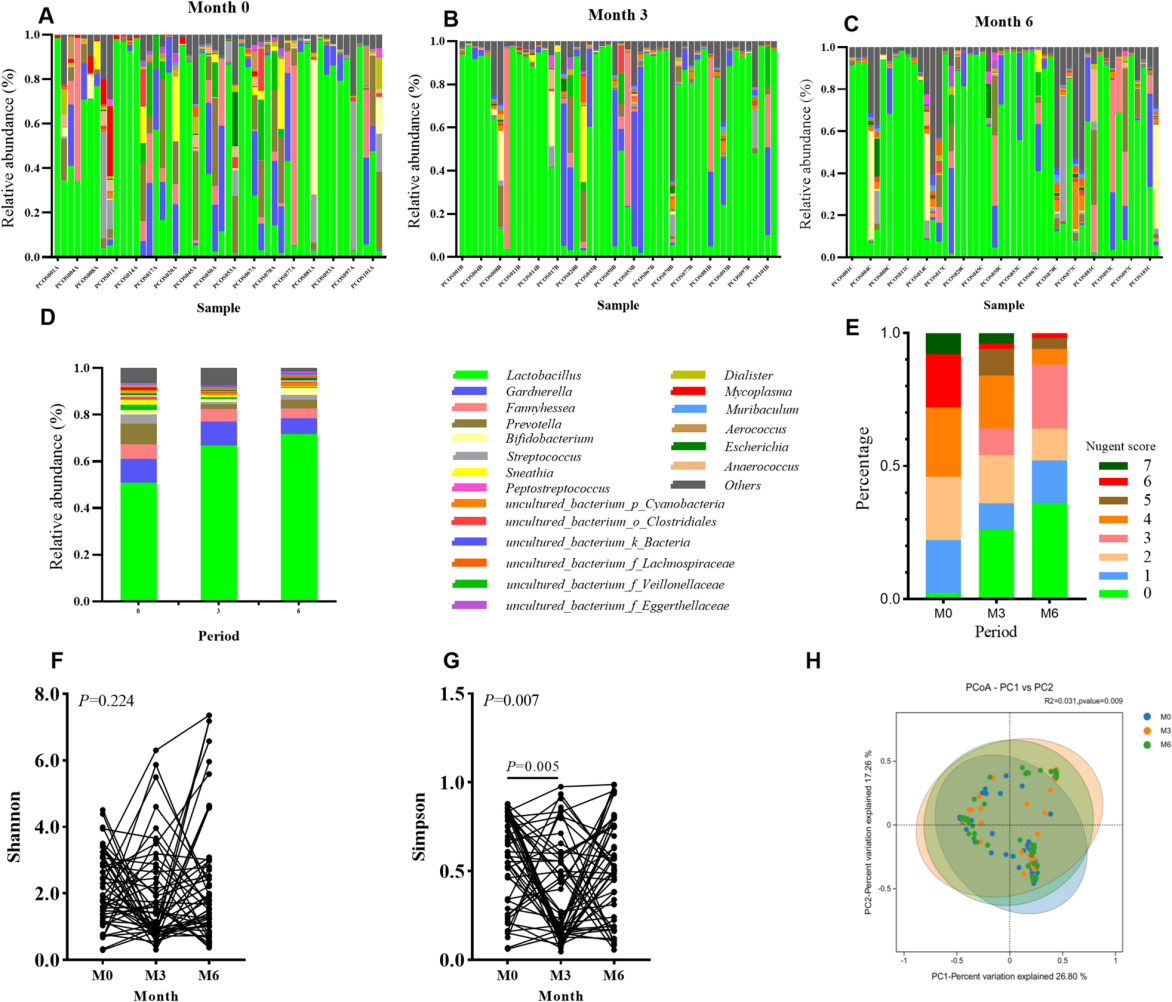
Longitudinal changes in the vaginal microbiome. **A**–**C** Histograms of the vaginal microbiota composition at baseline, month 3, and month 6. Genera are shown as different colors in the legend. **D** The average relative abundances of different genera. **E** Histogram of Nugent scores. **F** and **G** Shannon and Simpson index changes at different visits, *P*-values are from mixed-effect models. **H** PCoA plot based on Bray–Curtis distance. M0, M3, and M6 are baseline, month 3, and month 6 results

16S rRNA gene sequencing showed that the composition of the vaginal microbiota changed between baseline and months 3 and 6 (Fig. 2A, B, C). The relative abundance of *Lactobacillus* initially increased (green bars) from three to 6 months (Fig. 2D). Meanwhile, the proportion of patients with vaginal Nugent scores of 0–3 increased from 46% at baseline to 64% at 3 months and 88% at 6 months (Fig. 2E), indicating an improvement in the vaginal microenvironment. The PCoA plot with Bray–Curtis distance in Fig. 2H further confirmed that the differences of relative abundance of OTUs between the baseline and months 3 and 6 were statistically significant (PERMANOVA test, *P* = 0.009). Similarly, the PCoA plot with Jaccard distance presented a consistent result. (P < 0.001, Additional file 1: Fig. S1) However, no changes in the Shannon and Simpson indices were observed (Fig. 2F, G). Although there was a significant decrease in the Simpson index between baseline and month 3, the effect size was too small (*β* =  − 0.167, 95% CI: − 0.291, − 0.043), and many individual indices increased again from month 3 to month 6 (Fig. 2G). These results indicated that changes in the vaginal microbiome after treatment may not have been reflected by differences in the alpha-diversity indices.

### Association of hormone levels and the vaginal microbiome

**Fig. 3:**
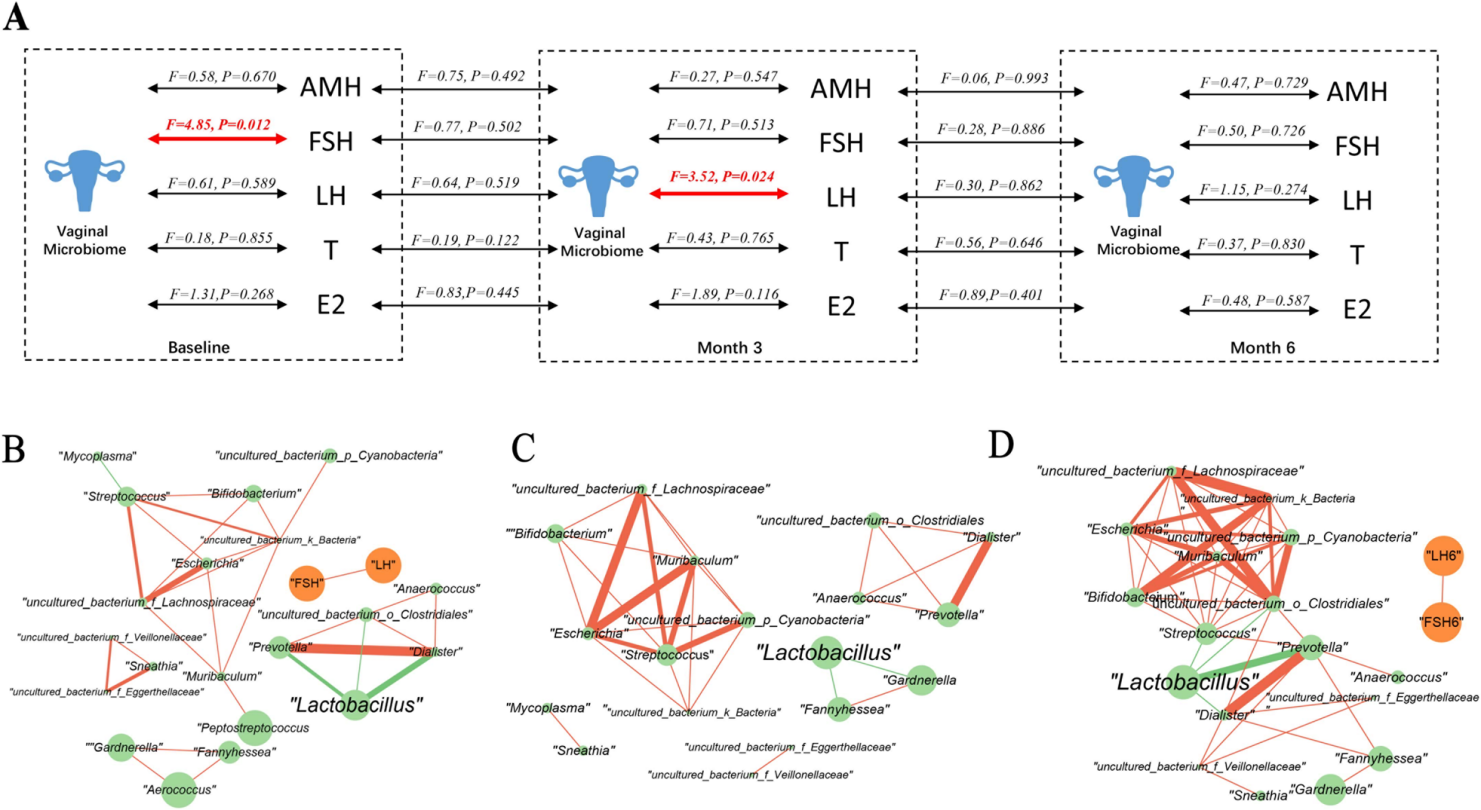
Association of hormone levels with vaginal microbiome. **A** Association of hormone level with the vaginal microbiome at different visits, all *P* values were adjusted by the Benjamini–Hochberg method. *F* and *P* values were inferred from the PERMANOVA analysis based on Bray–Curtis distance; **B**–**D** Network maps of the association of specific microbial genera and hormone level based on Spearman’s coefficients. Green circles indicate specific genera, orange circles indicate specific hormone indexes, and the circle diameter indicates relative abundance. Green lines indicate negative coefficients, red lines indicate positive coefficients, and line thickness indicates the coefficient value

To explore the possible impact of hormone levels on the vaginal microbiome, we analyzed the effects observed at different follow-up visits using PERMANOVA (Fig. 3A). At baseline, the vaginal microbiome was associated with FSH levels (*F* = 4.85, *P* = 0.012). At 3 months, it was associated with the existing LH level (*F* = 3.52, *P* = 0.024), but not with the baseline hormone levels (*P* > 0.05). At 6 months, the vaginal microbiome was not associated with existing or earlier hormone levels (*P* > 0.05). We did not find a significant relationship between hormone levels at the previous time point and the vaginal microbiota at subsequent time points (*P* > 0.05). Consequently, the association between the composition of the vaginal microbiome and hormonal changes may not be cause-and-effect.

Network analysis of different visits did not find an association between specific hormone levels and the abundance of specific genera (Fig. 3B–D). *Lactobacillus* abundance was negatively correlated with the abundance of *Prevotella* (*rho* =  − 0.71,* P* < 0.001) and *Dialister* (*rho* =  − 0.76,* P* < 0.001) at baseline (Fig. 3B) and negatively correlated with the abundance of *Fannyhessea* (*rho* =  − 0.68,* P* < 0.001) and *Gardnerella* (*rho* =  − 0.57,* P* = 0.004) at month 3 (Fig. 3C). At month 6, *Lactobacillus* was negatively correlated with the abundance of *Streptococcus* (*rho* =  − 0.61,* P* < 0.001), *Prevotella* (*rho* =  − 0.77,* P* < 0.001), and *Dialister* (*rho* =  − 0.64,* P* < 0.001) at month 6 (Fig. 3D).

### Latent class of vaginal microbiome and their affecting factors

**Fig. 4:**
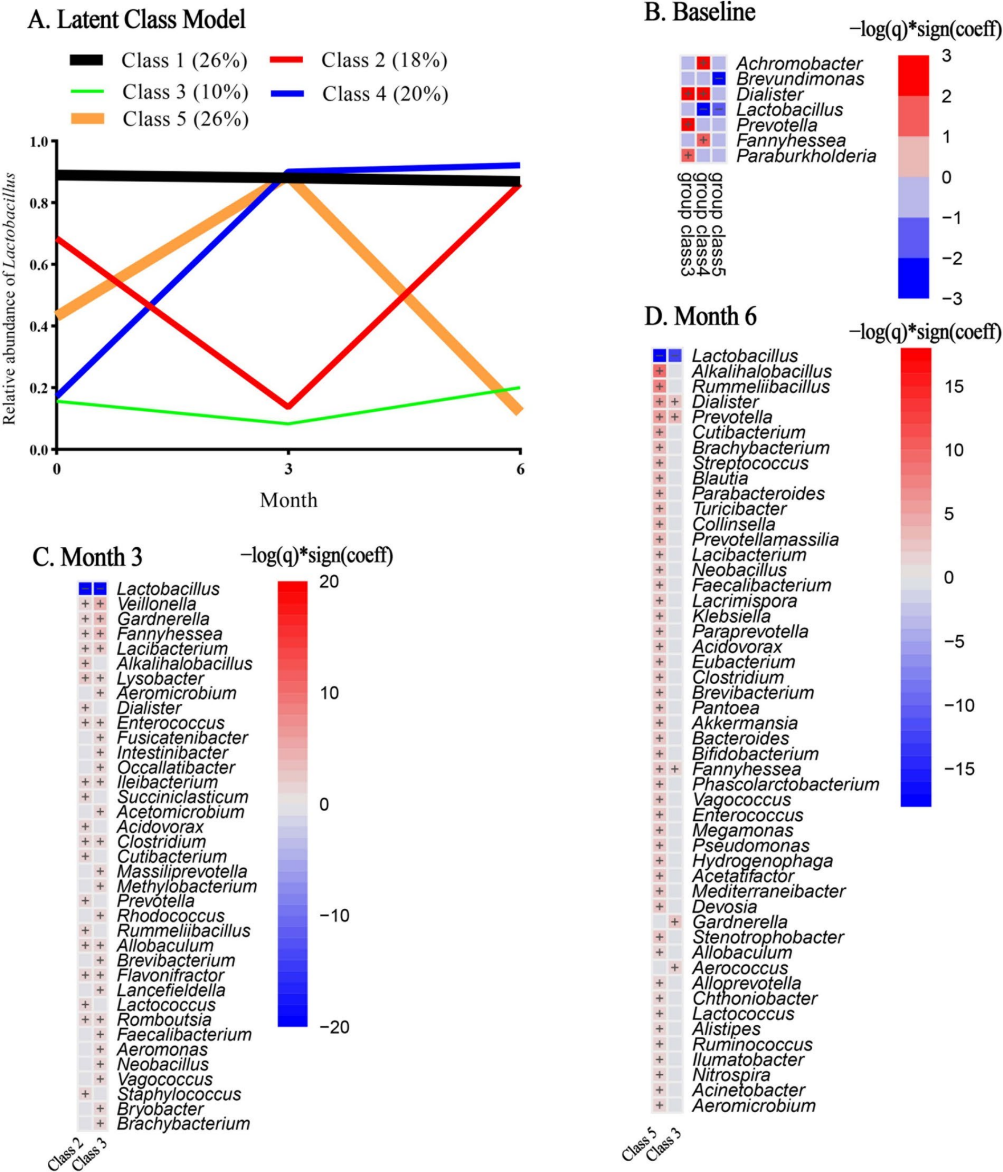
Latent class of the vaginal microbiome based on *Lactobacillus* relative abundance and their features. **A** Latent class trajectory model. Line thickness depends on the proportion of the class. **B**–**D** MaAsLin2 analysis at different visits, the reference group was class 1

Because *Lactobacillus* is the predominant genus in the vaginal microbiome, LCTM analysis was performed to determine the relative change in abundance. As shown in Fig. 4A, participants were divided into five classes. Class 1, with 26% of the participants, had a stable, high *Lactobacillus* abundance. In Class 2 (18%), *Lactobacillus* decreased and then increased. In Class 3 (10%), *Lactobacillus* abundance was low and stable. In Class 4 (20%), *Lactobacillus* abundance increased, and in Class 5 (26%), *Lactobacillus* abundance increased and then decreased. MaAsLin2 analysis identified genus biomarkers specific for different classes compared with class 1 at the three study visits (Fig. 4B–D). At baseline, class 4 was featured by *Achromobacter* and *Dialister*, class 3 was featured by *Prevotella*, *Dialister*, and *Paraburkholderia* (Fig. 4B). At month 3, class 2 and class 3 were all characterized by lower *Lactobacillus*. Class 3 was characterized by *Veillonella*, *Gardnerella*, and *Fannyhessea* (Fig. 4C). At 6 months, class 3 was characterized by *Dialister* and *Prevotella,* and class 5 by *Alkalihalobacillus* and *Rummeliibacillus* (Fig. 4D)*.* The LEfSe analysis with LDA threshold > 4.0 provided a similar result (Additional file 1: Fig. S2).

Multinomial logistic regression was used to explore the association between baseline hormone levels and microbiome classification, with adjusting for potential confounding factors (Table 2). Compared to class 1, an increased T level was associated with reduced risks for class 2 (OR = 0.03, 95% CI: 0.01–0.52 for 1 SD) and class 4 (OR = 0.10, 95% CI: 0.01–0.93 for 1 SD). This means that baseline T level might have an association with *Lactobacillus* change trajectory. The other indicators (FHS, LH, AMH, and E2) were not associated with trajectory classification (*P* values all > 0.05).

**Table 2:** Odds ratios of class inclusion for different baseline hormone levels

Hormonal indicators	Class 1	Class 2	Class3	Class 4	Class 5
FHS	Ref	1.28 (0.4,4.1)	0.58 (0.1,3.54)	0.3 (0.06,1.43)	1.31 (0.36,4.75)
LH	Ref	0.59 (0.21,1.7)	0.53 (0.07,4.14)	0.51 (0.14,1.88)	1.26 (0.49,3.24)
AMH	Ref	0.73 (0.26,2.07)	0.97 (0.27,3.47)	1.23 (0.47,3.23)	1.05 (0.37,2.98)
E2	Ref	1.07 (0.58,1.96)	1.22 (0.69,2.16)	0.88 (0.51,1.54)	1.2 (0.75,1.92)
T	Ref	**0.03 (0.01,0.52)**	0.19 (0.02,1.92)	**0.1 (0.01,0.93)**	0.94 (0.38,2.31)

All ORs were adjusted for age, marital status, education level, BMI, WHR, and baseline *Lactobacillus* levels. The hormone levels were *z*-transformed, the unit is one standard deviation

### Sensitivity analysis based on ASVs

When the sequences were denoised by the DADA2 method as ASVs, the main results were consistent. The differences of vaginal microbiome structure at three time points are visually presented through a PCoA plot (PERMANOVA test, *P* = 0.018, Additional file 1: Fig. S3). The microbial community structure of each sample was basically similar to that of the OTU table (Additional file 1: Fig. S4–S5). LCTM analysis also showed a 5-category grouping scheme, which is consistent with the above main results (Additional file 1: Fig. S6).

## Discussion

To the best of our knowledge, this is the first study to reveal longitudinal changes in the vaginal microbiome with oral contraceptive therapy in women of polycystic ovary syndrome. Overall, the vaginal microecology improved in the study participants, with an increase in the average relative abundance of *Lactobacillus*. There is no strong evidence linking hormone levels to changes in the vaginal microbiome. LCTM analysis identified five patterns of change based on *Lactobacillus* abundance of Lactobacillus. In some participants, *Lactobacillus* increased with treatment, but remained at a low level in others. It is not clear what caused this difference, but the baseline testosterone level was most likely involved. This finding emphasizes that more attention should be paid to changes in the vaginal microenvironment during PCOS treatment and that hormone testing should be considered when vaginitis is a concern.

Previous studies have not reported the effects of PCOS treatment on the vaginal microbiota, but the levels of female hormones are regarded as a potential factor affecting the vaginal microenvironment. Some longitudinal studies have tracked changes in the vaginal microbiome in relation to menstrual cycle [27]. In women without bacterial vaginitis, *Gardnerella vaginalis* abundance was shown to increase, and the abundance of *Lactobacillus* decreased during menses [28]. In menopausal women, *Lactobacillus* is always decreased and anaerobic taxa, including *Bacteroides* and *Mobiluncus*, are increased [29]. The changes are widely attributed to hormonal changes during menses or post-menopause [30]. There is evidence that hormone replacement therapy can restore vaginal *Lactobacilli* following menopause [3, 31], and vaginal estrogen therapy has been associated with increased *Lactobacillus* in the urine of postmenopausal women [32]. The current opinion is that estrogen promotes the maturation, proliferation, and accumulation of glycogen in vaginal epithelial cells and that glycogen is metabolized to lactic acid by *Lactobacillus* species, which creates an acidic environment that favors the growth of *Lactobacillus* [33]. However, as the predominant vaginal bacteria in more than 50% of postmenopausal women are Lactobacilli, [34, 35] simple estrogen-based hypotheses do not explain this phenomenon [30].

A focus on hormonal markers helps understand the relationship between hormones and the composition of the vaginal microbiota. AMH has been studied as an alternative to ultrasound for the PCOM evaluation of polycystic ovarian morphology [36]. AMH is a polypeptide belonging to the transforming growth factor-beta family. It is secreted by granulosa cells, inhibits the recruitment of primordial follicles in the resting oocyte pool, and suppresses FSH activity, contributing to ovulation disturbances [37]. PCOS patients always had increased AMH levels, and AMH levels were associated with many of its clinical symptoms. Previous studies have reported that AMH levels were negatively correlated with vaginal *Actinobacteria*, *Atopobium*, and *Gardnerella* abundance in patients with premature ovarian insufficiency [38]. We do not think it was a causal relationship because it was an observational case–control study. Our previous cross-sectional study indicated that neither the Shannon index nor the specific species were significantly associated with AMH levels in women with PCOS [8]. Increased testosterone in PCOS patients might result in acne and hirsutism, which cause a lot of distress and is significantly associated with changes in the vaginal microbiome in premature ovarian insufficiency patients [39]. T did not increase in all women with PCOS, and after hormone therapy, the effect of the difference on the vaginal microbiome is unknown.

However, we cannot conclude that the longitudinal change of the vaginal microbiome was attributed to oral contraceptive use in this study. According to ethical requirements, every patient received the education for a healthy lifestyle, and our results also reflect a decrease in their BMI. Previous observational studies had found that the vaginal microbiome is associated with BMI [40] and dietary habit [41], but the stronger evidence from randomized controlled trials (RCTs) is still lacking. Thus, the lifestyle changes would be the most important confounding factor in exploring the causal association between oral contraceptives and vaginal microbiome. Limited by ethical requirements, we cannot perform an RCT at this time, because these patients were all seeking medical intervention because of amenorrhea, only health education didn't meet their expectations. Further studies should use a bigger sample size cohort, then monitor the lifestyle change in detail, to explore the causes of changes in the vaginal microbiome. Meanwhile, it is interesting to observe the changes in vaginal microbiota among women with non-PCOS but using oral contraceptives for contraception, this result may help us to understand the effects without the impacts of physiological factors of PCOS.

In addition, there are many limitations to consider when interpreting the results. First, the sample size limited the statistical power, especially when the trajectory included five classes, which may have accounted for many results without statistical significance. Even so, we also think the findings would give some significant inspiration for clinical practice, because some differences were already seen at a small statistical power. A study with a larger sample is needed in the future. Second, annotations of specific species were not sufficient for 16S V3–V4 sequencing, thus the main analysis was performed at the genus level. Different *Lactobacillus* species may have different roles in the vaginal environment; in particular, *Lactobacillus iners* may have harmful effects [42]. Also, one species may have different biological functions in different environments. For example, *Gardnerella vaginalis* might produce more vaginolysin in *Lactobacillus iners*-dominated microbiomes [43]. These information might help us to understand longitudinal changes in the vaginal microbiome. Thus, analysis only at the genus level was not sufficient, although the current clinical Amsel test could not distinguish *Lactobacillus* species. Future studies should be conducted using a full-length sequencing approach to overcome this problem [44]. Third, some important confounding factors need to be considered in future studies, such as female age. The effects of age on hormones and vaginal microbiota are certainly present [45], but limited by our small sample size and limited age range, this study can not perform stratified analyses based on female age. Fourth, our study does not provide a good description of what happens to the vaginal microbiota in the first 3 months of treatment. Thus, a more intensive sampling time design, especially in the first 3 months, needs to be set up in the future. Lastly, the paucity of mechanistic studies hinders the interpretation of our results, thus, some in vitro or animal researches are needed to explore the underlying mechanisms between vaginal microbiome and PCOS.

Our current research is a prospective cohort study, which would help explore potential causality. However, we did not find a statistically significant relationship between hormone levels and vaginal microbiota at baseline and follow-up visits at 3 and 6 months. The vaginal microbiome and hormonal changes may be an adjoint rather than a causal association. All the patients benefited from oral contraceptive treatment. However, the exact cause of vaginal microbiome changes remains unknown from the current study. Interestingly, the baseline testosterone level seemed to have a potential impact on vaginal *Lactobacillus* change trajectories. This is the first report and deserves further study. In addition, we used bioinformatics and statistical analysis methods to explore the vaginal microbiome change trajectory and influencing factors in different parts of the body. For example, PERMANOVA focused on bacterial structure, whereas LCTM and logistic regression focused on *Lactobacillus* abundance. All of these methods guarantee the robustness of the results.

## Conclusions

Vaginal microbiome changes during oral contraceptive therapy in women with polycystic ovary syndrome. Overall, the abundance of *Lactobacilli* increased, but there were individual differences in the trajectory. Evidence for the effect of female hormone levels on the vaginal microbiome is insufficient. Future studies should further explore the vaginal microenvironment changes during the PCOS treatment period to provide more perspectives on PCOS and vaginal vaginitis treatment.

### Supplementary Information

**Additional file 1: Table S1.** The medians and quartiles for different hormone levels in different visits. **Figure S1.** PCoA plot based on Jaccard distance. **Figure S2.** Latent class of the vaginal microbiome based on *Lactobacillus* relative abundance and their features based on LEfSe analysis. **Figure S3.** PCoA plot based on Bray-Curtis distance with ASV table. **Figure S4.** The average relative abundances of different genera with ASV table. **Figure S5.** Histograms of the vaginal microbiota composition at baseline, month 3, and month 6 based on ASV table. **Figure S6.** Latent class of the vaginal microbiome based on Lactobacillus relative abundance with ASV table.

## Data Availability

The datasets presented in this study can be found in the online repositories. The names of the repository/repositories and their accession numbers (s) are available at NCBI PRJNA895690. Hong X, Wang B. Vaginal microbiome of PCOS women for 6 month follow-up Genome sequencing and assembly. NCBI, 2023. https://www.ncbi.nlm.nih.gov/bioproject/PRJNA895690/

## Abbreviations

AMH: Anti-Müllerian hormone
ASV: Amplicon sequence variant
BMI: Body mass index
CI: Confidence interval
E2: Estradiol
FSH: Follicle-stimulating hormone
LCTM: Latent class trajectory modeling
LH: Luteinizing hormone
MaAslin2: Multivariable association with linear models
OR: Odds ratio
OTU: Operational taxonomic unit
PCoA: Principal coordinate analysis
PCOS: Polycystic ovary syndrome
PERMANOVA: Permutational multivariate analysis of variance
RCT: Randomized controlled trial
T: Total testosterone
WHR: Waist-to-hip ratio
