# Supplementary material for: Change of the vaginal microbiome with oral contraceptive therapy in women with polycystic ovary syndrome: a 6-month longitudinal cohort study
Source: BMC Med. 2023 Dec 1;21:478. doi: 10.1186/s12916-023-03196-9 (PMC10693170; doi:10.1186/s12916-023-03196-9)
Supplement: Supplementary file 1 — Additional file 1: Table S1. The medians and quartiles for different hormone levels in different visits. Figure S1. PCoA plot based on Jaccard distance. Figure S2. Latent class of the vaginal microbiome based on Lactobacillus relative abundance and their features based on LEfSe analysis. Figure S3. PCoA plot based on Bray-Curtis distance with ASV table. Figure S4. The average relative abundances of different genera with ASV table. Figure S5. Histograms of the vaginal microbiota composition at baseline, month 3, and month 6 based on ASV table. Figure S6. Latent class of the vaginal microbiome based on Lactobacillus relative abundance with ASV table. [file 12916_2023_3196_MOESM1_ESM.docx]

**Contents**

**Table S1.** The medians and quartiles for different hormone levels in different visits.

**Figure S1.** PCoA plot based on Jaccard distance.

**Figure S2.** Latent class of the vaginal microbiome based on Lactobacillus relative abundance and their features based on LEfSe analysis.

**Figure S3**. PCoA plot based on Bray-Curtis distance with ASV table.

**Figure S4**. The average relative abundances of different genera with ASV table.

**Figure S5**. Histograms of the vaginal microbiota composition at baseline, month 3, and month 6 based on ASV table.

**Figure S6**. Latent class of the vaginal microbiome based on Lactobacillus relative abundance with ASV table.

**Table S1. The medians and quartiles for different hormone levels in different visits.**

|  | M0 | M3 | M6 |
| --- | --- | --- | --- |
| AMH, ng/mL | 8.41(6.17-11.63) | 4.44 (3.65-5.18) | 4.76 (4.31-5.41) |
| LH, IU/L | 12.04 (7.36-16.22) | 4.69 (3.09-5.83) | 4.46 (3.35-6.07) |
| FSH, mIU/mL | 6.65 (5.35-7.67) | 7.39 (6.30-8.45) | 7.32 (6.28-8.33) |
| T, ng/mL | 0.74 (0.57-0.94) | 0.45 (0.36-0.50) | 0.35 (0.32-0.41) |
| E2, pg/mL | 56.0 (44.0-88.0) | 38.0 (23.0-58.0) | 32.0 (23.5-54.0) |
| BMI, kg/m2 | 21.97 (19.95-25.38) | 21.16 (19.95-23.65) | 21.09 (20.07-22.55) |
| WHR | 0.82 (0.79-0.84) | 0.80 (0.78-0.81) | 0.78 (0.77-0.79) |


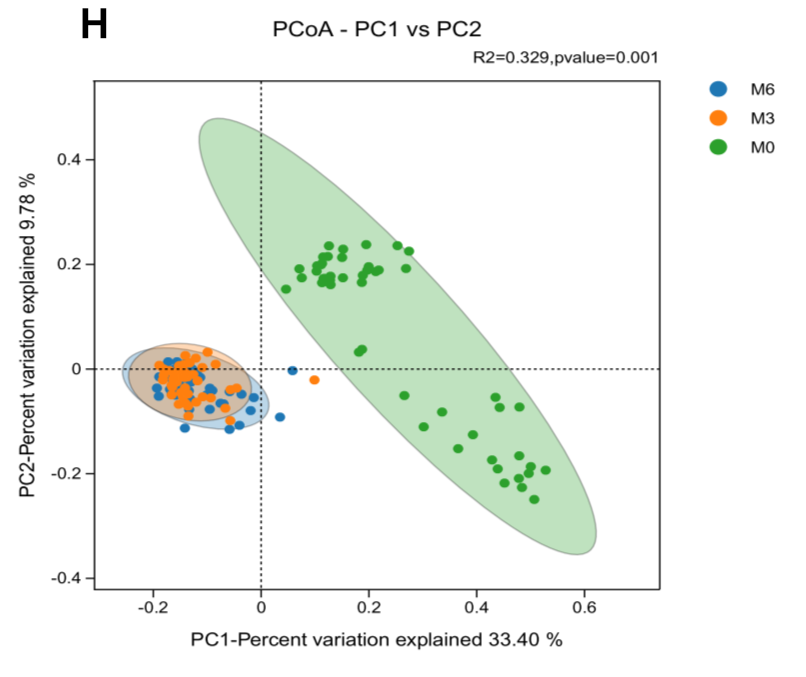


**Figure S1. PCoA plot based on Jaccard distance.** M0, M3, and M6 are baseline, month 3, and month 6 results.


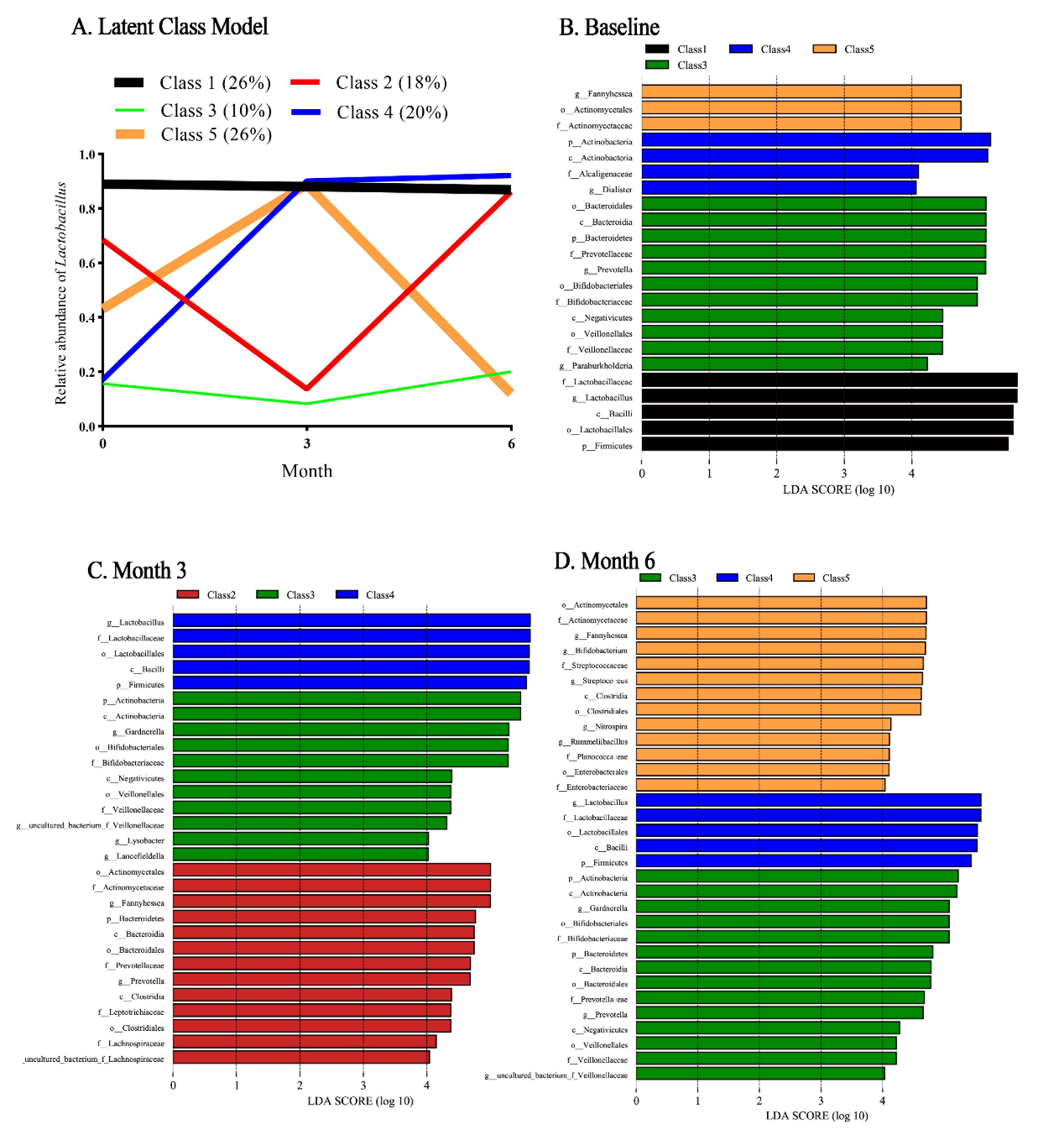


**Figure S2. Latent class of the vaginal microbiome based on Lactobacillus relative abundance and their features** **based on LEfSe analysis.** A, Latent class trajectory model. Line thickness indicates the proportion of the class. B–D, Lefse analysis of classes 3, 4, and 5 at different visits. The linear discriminant analysis (LDA) threshold was 4.0.

**
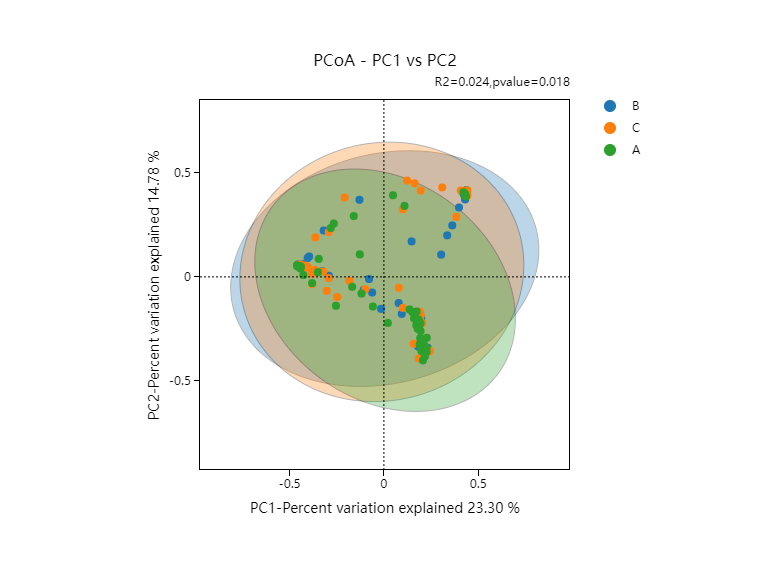
**

**Figure S3. PCoA plot based on Bray-Curtis distance with ASV table.** A, B and C are baseline, month 3, and month 6 results.


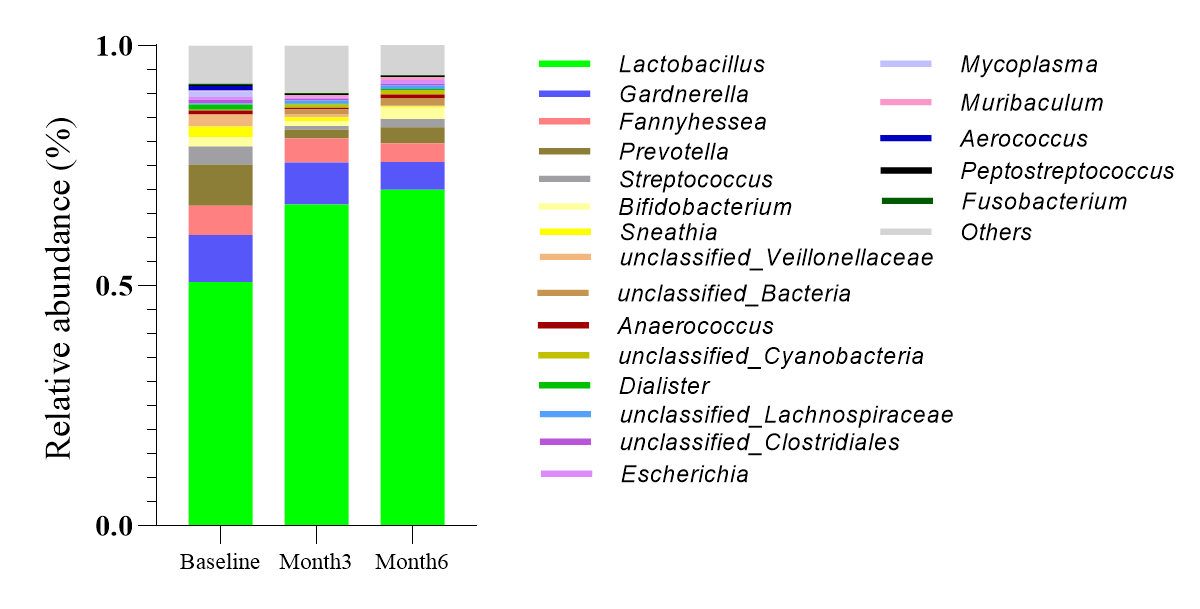


**Figure S4. The average relative abundances of different genera with ASV table.**

**A. Baseline**

**
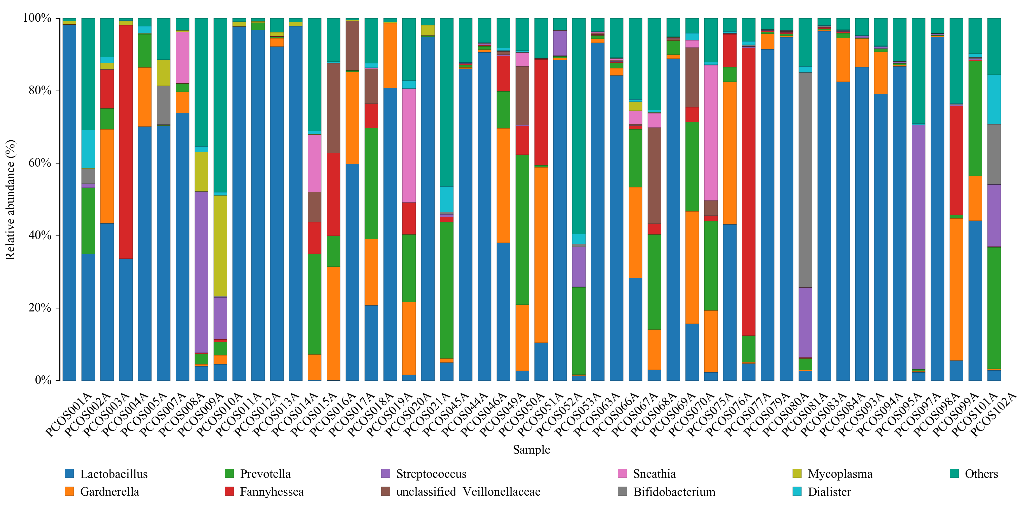
**

**B. Month 3**

**
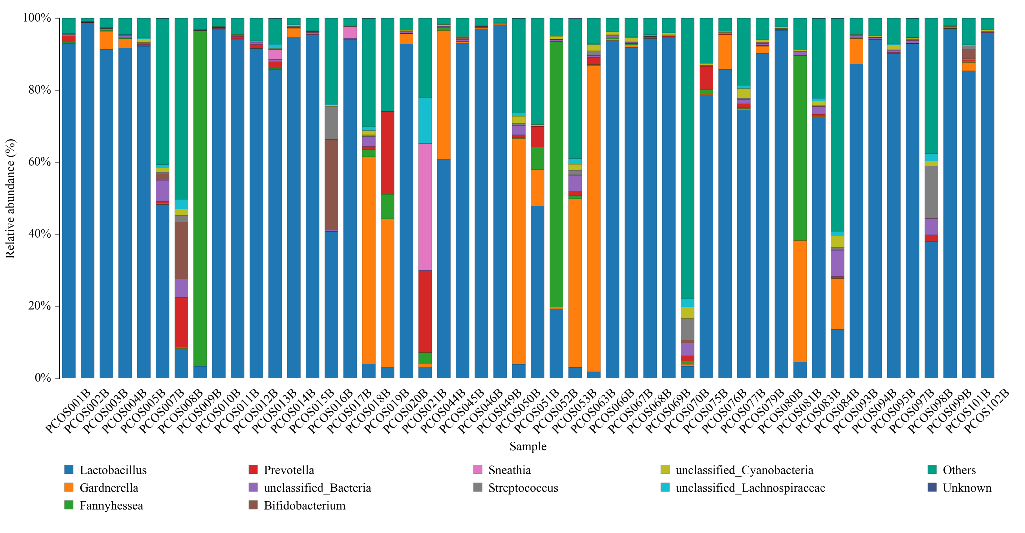
**

**C. Month 6**

**
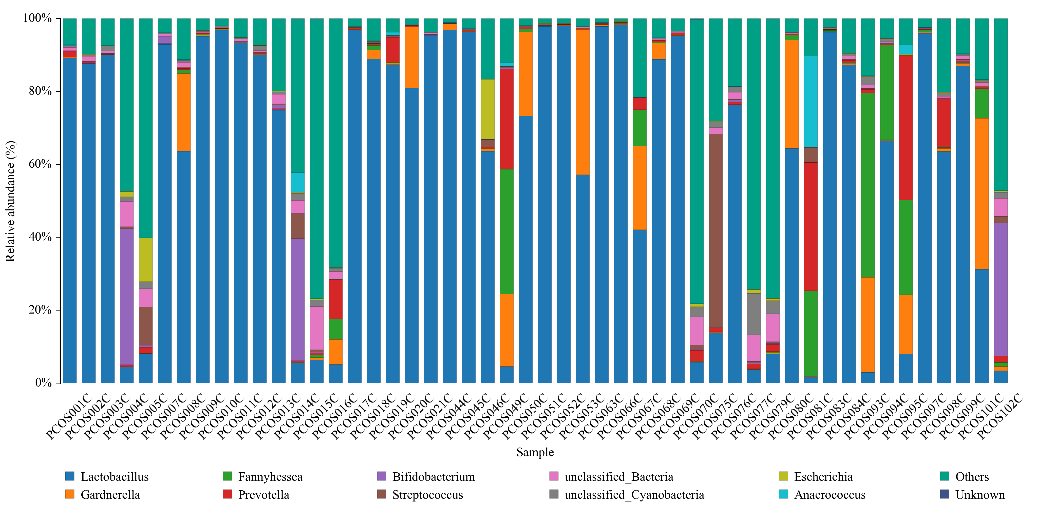
**

**Figure S5. Histograms of the vaginal microbiota composition at baseline, month 3, and month 6 based on ASV table.** Genera are shown as different colors in the legend.


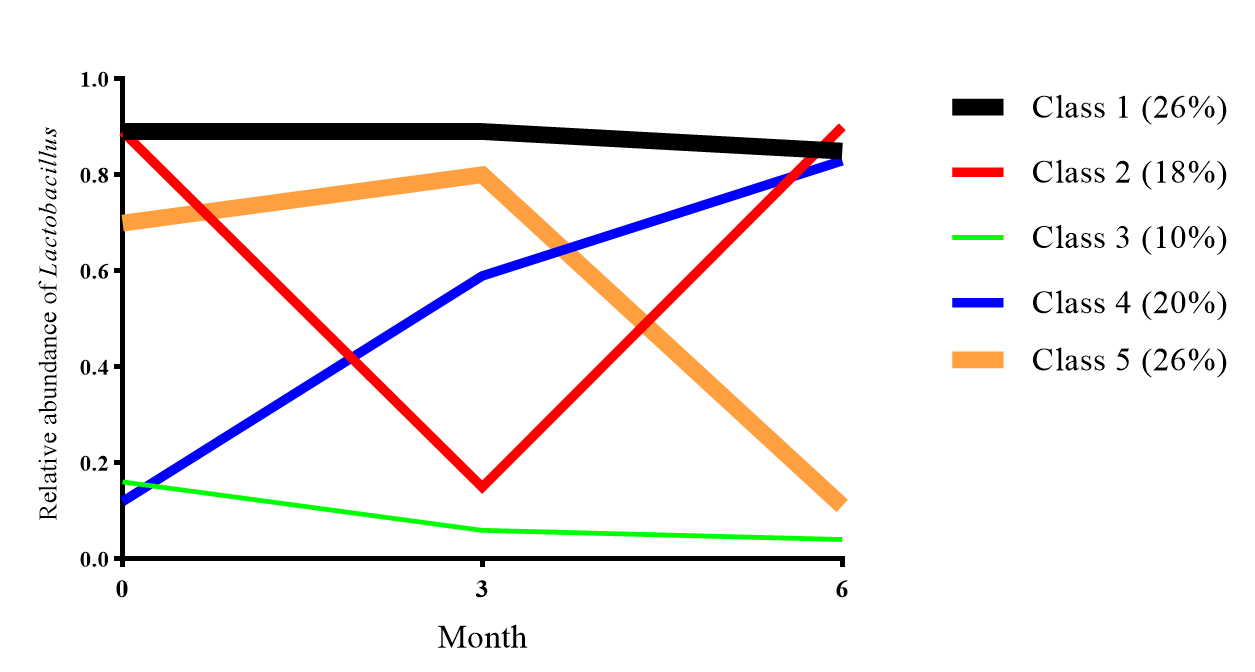


**Figure S6. Latent class of the vaginal microbiome based on Lactobacillus relative abundance with ASV table.**
